# Impact of Educational Level and Family income on Breast Cancer Awareness among College-Going Girls in Shillong (Meghalaya), India

**DOI:** 10.31557/APJCP.2020.21.12.3639

**Published:** 2020-12

**Authors:** Sutapa Biswas, Judita Syiemlieh, Roken Nongrum, Shashi Sharma, Maqsood Siddiqi

**Affiliations:** 1 *Cancer Foundation of India, Kolkata, West Bengal, India. *; 2 *Civil Hospital, Shillong, Meghalaya, India. *; 3 *National Institute of Cancer Prevention & Research, NOIDA, U.P, India. *

**Keywords:** Breast cancer awareness, BSE, risk factors, socio-demographic variables

## Abstract

**Background:**

Breast cancer (BC) is the most common cancer among women in India and shows an increasing trend. The mammography screening seems unfeasible as a public health service in India. Thus, breast self-examination (BSE), followed by clinical breast examination (CBE), is the affordable method to downstage BC. A cross-sectional study was conducted with senior school and college-going girls in Shillong (Meghalaya) to study the impact of girls’ academic level and family income on breast cancer knowledge and the prevalence of BC’s known risk factors in girls.

**Methods:**

A self-administered questionnaire was employed to collect relevant information. The data were analysed using statistical software SPSS version 22. The categorical data presented as frequency (%) and the comparison made using Chi-square or Fisher exact test.

**Results:**

(i) 78.2% girls knew about breast cancer, 19.2% of these were aware of BSE, and 22.9% of BSE knowing ever performed it (ii) Awareness of breast cancer and BSE, and its practice is significantly associated with their academic level and family income (iii) The consumption of alcohol beverages and physical activity of girls was positively associated with educational level and family income (iv) Body mass index (BMI) was weakly associated with family income with an insignificant relationship with academic level (v) oily food consumption related inversely with the level of education irrespective of family income (vi) there was a positive correlation between parents education and family income.

**Conclusions:**

The results show a severe lack of breast cancer knowledge in senior school and college-going girls under the survey. To spread community awareness, we suggest a public health policy-driven educational intervention through culturally relevant mass/social media on the risk factors of breast cancer and practice of BSE. It is also recommended that dedicated facilities be created for breast cancer early diagnosis in the public health system.

## Introduction

Breast cancer (BC) is the most common cancer among women in India. As per the latest Globocan, breast cancer constitutes 27.7 % of all female cases with an age-standardised incidence rates (ASR) of 24.7, and age-standardised mortality rate (ASR) of 13.4 (Globocan, 2018). Although India’s incidence rate is far below that of the Western world, where ASRs range between 80.0 and 95.0, the time trend analyses continually show an increasing annual percent change (APC) in breast cancer incidence (Ferlay et al., 2015; NCRP, 2016, Globocan, 2018). In a recent report, the rise of breast cancer between 1990 and 2016 was 39.1%, with the prevalent cases being 474 - 574 thousand (GBD Study, 2017). The increase of breast cancer incidence in India is commonly attributed to a perceptible change in urban women’s lifestyle, notably late marriages, delayed childbearing, low parity, and reduced physical activity (Khokhar, 2012; Youden et al., 2012, Porter, 2008). Also, the limited means of cancer awareness for women, inadequate early detection facilities, and a high cost of treatment of cancer further accentuate the problem, making breast cancer a critical health challenge in the country (Mittra, 2011; Gupta et al., 2015; Malvia et al., 2017; Sofi, 2019).

Breast cancer is a complex disease resulting from an interaction of environmental, genetic, and physiological factors; hence its primary prevention is currently unfeasible. The early diagnosis, therefore, is the most promising approach to a better prognosis. It is well known that most economically rich countries have succeeded in reducing breast cancer mortality by diagnosing it early using mammography screening (Shapiro et al., 1998). However, it is not feasible in India as a public health service or otherwise due to cost and logistic constraints (Smith et al., 2006; Okonkwo et al., 2008). Consequently, it becomes imperative to promote the affordable practice of breast self-examination (BSE) followed by clinical-breast examination (CBE), to downstage breast cancer (Anderson et al. 2015). Even though BSE per se is shown ineffective in reducing mortality rates (Thomas et al. 2002; Hacksaw and Paul, 2003), it is crucial in increasing breast health consciousness in women, and consequently facilitates downstaging breast cancer at first diagnosis (Miller and Baines, 2011; Dahlui et al., 2011). While intervention through BSE helps early detection of breast cancer in India, as shown by Gadgil et al., 2017, a deficient breast cancer awareness in women appears to be the critical obstacles to its universal acceptance in the country (Khokhar, 2010; Shankar et al., 2017).

The ensuing work is part of a study developing a module of community intervention in down-staging breast cancer in the North-Eastern India, which offers an ethnically and sociologically distinct society with a high incidence of breast cancer (NCRP, 2016). Also, India’s regional variations in lifestyle, being a multi-cultural society, make it essential to evaluate women’s breast cancer awareness and lifestyle practices before initiating a population-based intervention. Keeping this in view, we present here the results of the first cross-sectional study assessing the level of breast cancer awareness among senior-school and college-going girls in Shillong (Meghalaya), in North East India. The study also evaluates the impact of girls’ current academic status and family income on the extent of breast cancer and BSE awareness and practice. These variable’s relationship with the prevalence of known lifestyle-associated breast cancer risk factors in girls was also studied.

## Materials and Methods

*Study setting and Design*

Senior school and college-going girls from 13 randomly chosen educational institutions in urban and peri-urban areas in Shillong (Meghalaya), India, participated in a cross-sectional descriptive study. A specially constructed and pretested self-administered questionnaire in English collected the required information. Of the 2023 students who were sensitized with the survey’s objective, 1916 (response rate - 94.7%) students volunteered to self-answer the questionnaire.

*Study Questionnaire*

The questionnaire consisted of six sections, the first being on socio-demographic details of the participant related to address, age, religion, current educational status. The second part contained questions on reproductive history like age at menarche, marital status etc. Part three had queries on anthropometry, including the height, weight, waist size. In section four, respondents answered questions on alcohol drinking and other habits. Part five consisted of questions assessing their knowledge of cancer in general and breast cancer, its risk factors, symptoms, and whether a close relative in the family is affected by it. The sixth part examined the respondent’s knowledge and practice of BSE and the attitude towards learning BSE and spreading breast cancer awareness in the community. The last section consisted of questions on physical exercise and the consumption of high fat and oily food.

*Statistical Analysis*

The data were entered in Microsoft Excel and analysed using statistical software SPSS (IBM Corp Ltd. Armonk NY, USA) version 22. The categorical data presented as frequency (%) and the comparison was made using Chi-square or Fisher exact test as and where appropriate. Data were analysed for the association between categorical variables by Pearson Chi-square.

## Results

*Demographic Characteristics*

Age and Religion: Nineteen hundred and sixteen (94.7%) students completed the questionnaire out of 2,023 (Table-1). The mean age of the respondents was 19.9 years (95% CI, 19.89 – 20.03). The predominant age group was between 18 and 25 years, with most of them between 18 and 21 years (1633; 85.2%), and 275 (14.3%) between 22 and 25. Only two respondents were above 30 years. The respondents were largely Christians (1463; 76.4%) with 281 Hindus (14.7%), 26 Muslims (1.4%), 6 Sikhs (0.3%) and 140 Others (7.3%). 

Education of Girls: It is quite a broader range of students with a majority of college-going girls doing graduation in arts, social science, commerce, and business administration (1136; 59.3%) and 399 (20.8 %) from science classes; the ratio between these two groups being 3:1. Three hundred and nine girls (16.1%) were completing twelfth year of schooling, and Seventy-two girls (3.8%) were doing post-graduate courses or higher education. 

Education of Parents: The highest education level of fathers of 1144 girls (59.7%) was up to school level, whereas 478 (24.9%) were graduates, 216 (11.2 %) post-graduates and above, and 78 (4.0 %) had vocational education. Mothers of 1320 girls (68.9 %) had school education, 359 (18.7%) were graduates, 157 (8.2%) post-graduate and above, and 80 (4.2%) had alternate or vocational education.

Family Income: As shown in Table 1, the family income of 409 girls (21.3 %) was less than Indian Rupees (INR) 5000 per month. Eight hundred five (42.0 %) belonged to families whose income was between INR 5001 and 15,000, 397 (20.7 %) were from INR 15,001 to 25,000, and 202 (10.5 %) girls came from families with an income of INR 25,001 to 50,000. One hundred and three girls (5.4%) belonged to higher-income families, i.e., INR 50,000 and above per month. 

*Reproductive History*

Menarche: The mean age of menarche among the respondents was 13.35 (95% CI, 13.29 – 13.41). As shown in Table 1., One hundred and ten girls (5.7%) had their menarche between 9 and 11 years, 1462 (76. %) had between 12 and 14 years, 321 (16.7%) from 15 to 16 years, and 6 girls had their menarche at 17 years or later.

Married Girls: There were only 20 married girls in the cohort with a mean age at marriage of 20.2 years (95% CI; 20.87–19.52). Of these, 12 girls had one child, 2 having two each, and one had more than two children. They had a mean age of 20.76 years (95% CI; 21.42 – 20.10) at first childbirth, and thirteen girls breastfed their children for a duration ranging 2-24 months. Only 10 (50.0%) used contraceptives, whereas 3 (15.0%) of the married girls were unaware of birth-control measures.

*Awareness of Cancer and Breast Cancer*

The survey instrument contained questions to assess girls’ knowledge of cancer in general and breast cancer, including the risk factors, signs, symptoms, BSE and its practice, etc. Of the 1916 girls who participated in the study, 1844 (96.2%) girls had heard of cancer, 63.3.% also knew that the disease is managed better when detected early. Though the primary source of cancer information was friends and relatives (51.2%), 26.7% of girls learned about cancer from TV/Radio and 23.8% from newspapers. Health awareness programmes accounted for only 21% (Table 2). 

On the other hand, 78.2% respondents (1497) knew about breast cancer, and only 35.9 % of these (687) were confident that breast cancer prognosis is good if detected early, and others were either unsure or believed that it was not curable (309; 20.6 %). Only 19.23% of breast cancer knowing girls, and 288 (15.0%) of all the respondents, had ever heard of breast self-examination (BSE), and 66 (22.9%) BSE knowing girls had ever performed it, amounting to only 3.5% of the girls in the survey. A divided response was received from girls when asked about the possible lifestyle or behavioural risk factors of breast cancer. 18.8% said genetic factors, 6.0% attributed it to late marriage, 5.5% ascribed it to late pregnancy, and 6.1% to obesity as a significant risk factor for breast cancer. 34.5% of respondents considered Pain and 42.4% a lump in the breast and 19.1% nipple discharge, and 14.2% girls considered retracted nipple and 12.2 % dimpling on the chest as significant signs & symptoms of breast cancer. There were questions to gauge girls’ attitudes towards learning BSE and for propagating it in the community. Given an opportunity, 93.6% of girls expressed their desire to learn BSE. In contrast, only 46.8% were willing to volunteer, creating community awareness of breast cancer and BSE to promote early cancer detection.

*Modifiable Lifestyle Risk Factors*

Physical Activity and Consumption of Oily food: Several questions were included in the questionnaire to collect information on lifestyle practices known to increase breast cancer risk. As shown in Table 4., 86 girls (4.5%) did a planned physical activity for 1-hour every day, 185 (9.7%) did for 30 minutes. There were 271 girls (14.1%) who did a physical exercise for at least 15 minutes daily, whereas 761 (39.7%) reported occasional physical activity, and 613 girls (32.0%) did no intentional physical exercise. High fat and oily food were taken more than once daily by 278 girls (14.5%), 547 girls (28.5%) once daily, and 709 girls (37.0%) had it occasionally, whereas 382 girls (19.9%) stated that they rarely consumed oily food. 

Alcohol and Body Mass Index (BMI): Two hundred and twenty-five (11.7%) respondents stated to be ever consumers of alcoholic beverages (Table 4), with most of them declaring to be an occasional drinker, and only 3 took alcoholic drinks regularly. As shown in Table 1, the BMI was calculated based on respondent’s height and weight information. The mean BMI of 1916 girls was 21.01 Kg/m^2^ (95% CI; 20.81 – 21.21), with a minimum of 11.46 and a maximum of 45.86 Kg/m^2^. Six hundred and sixty-eight girls (34.9%) were underweight with a BMI of <18. Eight hundred and seventy-six girls (45.7%) were having average weight with a BMI of 18.5-24.9 Kg/m^2^, and 297 (15.5%) were overweight or belonged to the Class I of Obese (BMI 25.0-29.9 Kg/m^2^). There were 14 girls (0.7%) in Class II of Obesity within a BMI rage 30.0-39.9 Kg/m2, and 61 girls (3.2%) had Class III Obesity with a BMI of >40 Kg/m^2^.

*Association of Awareness of BC, BSE and its practice with Educational level of girls and Family Income*

The Chi-square statistic was employed to study the association of girls’ educational level and family monthly income with the awareness of breast cancer, BSE, and BSE’s practice. The groupings of the educational level of girls and family income are shown in Table 1. It is evident from the data presented in Table 3 that the girls’ awareness of breast cancer is significantly associated with the educational level of girls [*χ*^2^ (3; N=1916) 61.553; p<.001] and the monthly income of the family [*χ*^2^ (4; N=1916) 47.655 p<.001]. The awareness of BSE [[*χ*^2^(3; N=1916) 112.077; p<0.001] and its practice [[*χ*^2^ (3; N=1916) 22.095; p<0.001] is also positively associated with education level of girls and the monthly income of the family [[*χ*^2^ (4; N=1916) 39.846, p<0.001 and [*χ*^2^ (4; N=1916) 33.037, p<0.001]. 

*Association of Risks Associated Lifestyle practices with Educational level of girls and Family Income*

Chi-Square test was also used to understand the association between the frequency of physical activity, consumption of oily foods, the habit of alcohol drinking, and girls’ BMI with their educational level and family income. Planned physical activity was evaluated in 5 extents, i.e., 60, 30, and 15 minutes, occasional, and never. The level of physical activity is significantly associated with the educational level of girls [[*χ*^2^ (12; N=1916) 62.149, p<0.001], and economic background [[*χ*^2 ^(16; N=1916) 56.621, p<0.001]. In contrast, the frequency of taking oily food, is inversely related to the educational level; This inverse relationship was statistically significant [[*χ*^2^ (9; N=1916) 68.517, p<.001]. However, there was a much weaker or insignificant association with the family income [[*χ*^2^ (12; N=1916) 24.074, P=0.020]. Of the 1916 girls, 225 (11.7%) admitted being ever consumers of alcohol. The Chi-Square test showed a significantly positive relationship between the consumption of alcoholic beverages and the education of girls [[*χ*^2^ (6; N=1916) 45.314, p<0.001], and also the total income of the family they came from [[*χ*^2 ^(8; N=1916) 35.508, p<0.001]. The body mass index (BMI) reflects the weight in relation to height. It thus measures obesity, which is a vital risk determinant of several non-communicable diseases, including breast cancer. As shown in Tables 1 and 4, 19.4% of the respondent girls are clinically obese, having a BMI > 25.0. Although less percentage of obese girls are seen in the group of PG girls compared to girls in school, the association of BMI with girls’ education level when examined using the Chi-square test, was not significant [[*χ*^2^ (6; N=1916) 12.835, P = 0.046]. The association of family income with BMI is weakly significant [[*χ*^2^ (8; N=1916) 24.672, P = 0.002] (Table 4).

The survey instrument contained questions on the highest educational qualification of parents. As shown in the Table 5., there is a positive association of father’s [[*χ*^2^ (12; N=1872) 353.705, p<0.001] and mother’s educational status [[*χ*^2 ^(12; N=1872) 353.705, p<0.001] with family income when checked by Chi-square test. 

**Table 1:** Socio-Demographic Characteristics of Girls

Characteristics	n (%)	
Age in Years		
18-21	1633	85.2
22-25	275	14.3
26-33	8	0.4
Religion		
Christian	1463	76.4
Hindu	281	14.7
Muslim	26	1.4
Sikh	6	0.3
Others	140	7.3
Education		
School	309	16.1
Graduate (Arts)	1136	59.3
Graduate (Science)	399	20.8
Post-Grad and above	72	3.8
Father's Education		
School	1,144	59.7
Graduate	478	24.9
Post-Grad and above	216	11.2
Others	78	4
Mother's Education		
School	1320	68.9
Graduate	359	18.7
Post-Grad and above	157	8.2
Others	80	4.2
Family Income (per m) INR		
< 5,000	409	21.3
5,001 - 15,000	805	42
15,001 - 25,000	397	20.7
25,001 - 50,000	202	10.5
> 50,000	103	5.4
Marital Status		
Unmarried	1,896	98.9
Married	20	0.1
Age at Menarche (years)		
9-11	110	5.7
12-14	1462	76.3
15-16	321	16.7
>16	23	1.2
Body Mass Index (BMI)		
Underweight (<18.5)	668	34.9
Normal weight (18.5-24.9)	876	45.7
Class I Obesity (25.0-29.9)	297	15.5
Class II Obesity (30.0-39.9)	14	0.7
Class III Obesity (>40)	61	3.2

**Table 2:** Girls’ Awareness on Cancer and Breast Cancer

Questions	Yes		No. of Respondents
	n	%	
a. Knowledge on Cancer			
Heard about cancer	1844	96.2	1915
Can cancer be managed better if detected early	1211	63.3	1913
b. Initial source of information on Cancer			
Friends/Relatives	964	51.2	1883
TV/Radio	503	26.7	1884
Newspaper	448	23.8	1884
Health awareness programs	395	21	1884
c. Knowledge on Breast cancer / BSA			
Heard about breast cancer	1497	78.2	1914
Can breast cancer be managed better if detected early	687	35.9	1916
Have you heard about Breast Self-examination (BSA)	288	15	1916
Ever conducted Breast Self-examination (BSA)	66	3.5	1916
d. Factors increasing risk of Breast cancer			
Smoking	283	17.7	1597
Breast-feeding	284	17.8	1596
Alcohol	149	9.3	1596
Late marriage	96	6	1596
Genetic Factors	300	18.8	1596
Late pregnancy	88	5.5	1596
e. Symptoms of Breast Cancer			
Pain	551	34.5	1598
Lump	677	42.4	1598
Discharge from Nipple	306	19.1	1598
Retracted nipple	227	14.2	1598
Depression / Dimpling of skin	195	12.2	1597

**Table 3:** Association of Breast Cancer Awareness, BSE Awareness and BSE Practice with Educational level of Girls and Family Income

Education & Economic level	No. of Girls n (%)	Breast Cancer Awareness Yes (%)	BSE Awareness Yes (%)	BSE Practice Yes (%)
Girls’ Education				
School	309 (16.1)	228 (73.8)	20 (6.5)	5 (1.6)
Graduation (Arts)	1136 (59.3)	840 (73.9)	129 (11.4)	29 (2.6)
Graduation (Sci)	399 (20.8)	365 (91.5)	124 (31.1)	28 (7.0)
Post-Graduate & above	72 (3.8)	64 (88.9)	15 (20.8)	4 (5.6)
Chi-Square		χ2 (3; N=1916) 61.553; p<0.001	χ2 (3; N=1916) 112.077; p<0.001	χ2 (3; N=1916) 22.095; p<0.001
Family Income (monthly) INR				
< 5,000	409 (21.3)	272 (66.5)	33 (8.0)	16 (3.9)
5,001 – 15,000	805 (42.0)	644 (80.0)	113 (14.0)	15 (1.9)
15,001 – 25,000	397 (20.7)	319 (80.4)	67 (16.9)	9 (2.3)
25,001 – 50,000	202 (10.5)	178 (88.1)	47 (23.3)	19 (9.4)
> 50,001	103 (5.4)	84 (81.5)	28 (27.2)	7 (6.8)
Chi-Square		χ2 (4; N=1916) 47.655 p<0.001	χ2 (4; N=1916) 39.846, p<0.001	χ2 (4; N=1916) 33.037, p<0.001

INR, Indian Rupee

**Table 4:** Association of Lifestyle Risk Factors with Educational Level of Girls and Family Income

Education & Economic level	Number Of Girls n (%)	Physical Exercise - n / (%)					Fried/Oily food – n / (%)				Alcohol	BMI [n (%)]		
		60 min daily	30 min daily	15 min daily	Occa- sional	Never	Once daily	>Once daily	Occa- sional	Rarely	Yes (%)	Under Weight <18.5	Normal Weight 18.5-24.9	Obese Class I-III > 25.0
Girl’s Education		n=86	n=185	n=271	n=761	n=613	n=547	n=278	n=709	n=382	n=225	n=668	n=876	n=372
School	309 (16.1)	10 (3.2)	32 (10.3)	54 (17.5)	106 (34.3)	107 (34.6)	64 (20.7)	49 (15.8)	106 (34.3)	90 (29.1)	32 (10.3)	116 (37.5)	134 (43.4)	59 (19.1)
Graduate (Arts)	1136 (59.3)	57 (5.0)	103 (9.0)	155 (13.6)	409 (36.0)	412 (36.3)	367 (32.3)	164 (14.4)	371 (32.6)	234 (20.6)	130 (11.4)	377 (33.2)	514 (45.2)	245 (21.6)
Graduate (Science)	399 (20.8)	15 (3.7)	40 (10.0)	55 (13.8)	206 (51.6)	83 (20.8)	97 (24.3)	56 (14.0)	195 (48.9)	51 (12.8)	37 (9.3)	150 (37.6)	190 (47.6)	59 (14.8)
Post-Graduate & above	72 (3.8)	4 (5.5)	10 (13.9)	7 (9.7)	40 (55.6)	11 (15.2)	19 (26.4)	9 (12.5)	37 (51.4)	7 (9.7)	26 (36.1)	25 (34.7)	38 (52.4)	9 (12.5)
Chi-square	χ^2^ (12; N=1916) 62.149, p<0.001						χ^2^ (9; N=1916) 68.517, p<0.001				*	χ^2^ (6; N=1916) 12.835, P = 0.046		
Family Income (monthly) INR														
< 5,000	409 (21.3)	14 (3.4)	23 (5.6)	68 (16.6)	135 (33.0)	169 (41.4)	119 (29.1)	51 (12.5)	142 (34.7)	97 (23.7)	23 (5.6)	130 (31.8)	169 (41.3)	110 (26.90)
5,001 – 15,000	805 (42.0)	36 (4.5)	80 (9.9)	95 (11.8)	322 (40.0)	272 (33.8)	219 (27.2)	118 (14.6)	289 (35.9)	179 (22.3)	92 (11.4)	294 (36.6)	363 (45.0)	148 (18.4)
15,001 – 25,000	397 (20.7)	22 (5.5)	48 (12.0)	64 (16.1)	160 (40.3)	103 (25.9)	113 (28.5)	61 (15.4)	153 (38.5)	70 (17.6)	52 (13.0)	141 (35.5)	191 (48.1)	65 (16.4)
25,000 – 50,001	202 (10.5)	9 (4.4)	21 (10.4)	35 (17.3)	91 (45.0)	46 (22.8)	63 (31.2)	28 (13.8)	84 (41.6)	27 (13.4)	38 (18.8)	68 (33.7)	107 (53.0)	27 (13.3)
> 50,000	103 (5.4)	5 (4.8)	13 (12.6)	9 (8.7)	53 (51.4)	23 (22.4)	33 (32.0)	20 (19.4)	41 (39.9)	9 (8.7)	20 (19.4)	35 (34.0)	46 (44.6)	22 (21.4)
Chi-square	χ^2^(16; N=1916) 56.621, p<0.001						χ^2^ (12; N=1916) 24.074, P=0.020				**	χ^2^ (8; N=1916) 24.672, P = 0.002		

INR, Indian Rupee; *χ^2^ (6; N=1916) 45.314, p<0.001; ** χ^2^(8; N=1916) 35.508, p<0.001

**Table 5:** Association of Parent’s Educational status with Family Income

Family Income (monthly) INR)	Father’s Education [n (%)]					Mother’s Education [n (%)]				
	School	Graduation	PG and above	Others	Total	School	Graduation	PG and above	Others	Total
< 5000	320 (81.8)	37 (9.4)	15 (3.8)	19 (4.8)	391	330 (85.7)	26 (6.7)	13 (3.4)	16 (4.1)	385
5000-15000	548 (69.4)	174 (22.0)	58 (7.3)	10 (1.2)	790	608 (77.1)	123 (15.6)	47 (5.9)	10 (1.3)	788
15001 -25000	188 (47.9)	140 (3.5)	63 (16.0)	1 (0.2)	392	248 (63.3)	103 (6.7)	39 (9.9)	2 (0.5)	392
25001 - 50000	69 (34.8)	88 (44.4)	37(18.7)	4 (2.0)	198	100 (50.0)	64 (31.5)	34(17.0)	2 (1.0)	200
> 50000	19 (18.8)	39 (38.6)	43 (42.5)	0 (0.0)	101	34 (33.6)	43 (42.6)	24 (23.8)	0 (0.0)	101
Total	1144 (61.1)	478 (25.6)	216 (11.5)	34 (1.8)	1872	1320 (70.7)	359 (19.3)	157 (8.4)	30 (1.6)	1866
Chi-square	χ^2^(12; N=1872) 353.705, p<0.001					χ^2^(16; N=1866) 229.135, p<0.001				

INR, Indian Rupee

## Discussion

The results presented here show an innate deficiency of breast cancer knowledge in senior school and college-going girls in Shillong. While nearly all the girls were aware of cancer, only three-fourths had ever heard of breast cancer. The primary source of cancer information was friends and relatives, while TV/Radio, newspapers, and health awareness programs contributed little. The knowledge about breast cancer’s risk factors and its signs and symptoms was highly ambiguous, and only 83.3% of girls attempted these questions. Our results indicate that breast cancer’s lack of understanding extends to BSE’s knowledge and its practice among girls too. Only 19.2% of breast cancer knowing girls ever heard of breast self-examination, and 22.9% of those had ever performed it, amounting to 3.5% of girls out of the total who participated in the survey (Table 2). These results are congruent with earlier reports from India, showing a severe deficiency of breast cancer awareness among different age groups of women from diverse socioeconomic and geographical backgrounds (Yadav and Jaroli, 2010; Gupta et al., 2015; Gangane, 2015).

Several modifiable lifestyle factors are known to be strongly associated with increased breast cancer risk. These include consuming alcohol, lack of physical exercise, being overweight or obese in adulthood, and a high intake of saturated fat (WCRF and AICR, Continuous Update Project, 2018, ACS: Breast Cancer Facts and Figures, 2019). Our results show that 11.7% of girls were ever consumers of alcohol. Although our report seems higher than the one reported by Chaturvedi et al., (2003), showing that only 3.2% of females consume alcohol in Meghalaya and upper Assam, it looks appropriate in view of a recent study from neighbouring Assam state reporting 22.2% ever consumers of alcoholic drinks among school-going boys and girls (Bhagabati et al., 2013). It also confirms the perception of increased use of alcoholic beverages among youth in India. Obesity is a known risk factor of BC for post-menopausal women, especially those who gain 25 Kgs or more from the age of 18 (Heather Eliassen, 2006). Our results show that while a large majority of girls were normal or underweight, having a mean BMI of 21.01 Kg/m^2^ (95% CI; 20.81 – 21.21), 15.5% girls were overweight or having Class 1 obesity (25.0-29.9 Kg/m^2^), and 3.9% were obese of Class II and III having a BMI of 30 Kg/m^2^ and above. Although these the data are within the range of the average national figure of 17.3% of overweight and 5.9% of obese urban women in India (Gouda and Prusty, 2014), the fact that 3.2% girls have Class III Obesity with a BMI of >40 Kg/m^2^ is indeed worrisome and needs public health attention. Since obesity in adulthood is an established yet preventable risk of breast cancer, it must form an essential component of every educational intervention among young girls.

Other known risk factors of breast cancer are those associated with reproductive life, including early menarche, late menopause, and low parity (Maria et al., 2014). With the restricted age-group of predominantly unmarried girls in the present survey, we could only profile the age at menarche shown in Table 1. Details about 20 married girls are in Results. Our data shows a mean age at menarche was 13.35 years (95% CI, 13.29 – 13.41), which is slightly lower than the national average of 13.76 years (Pathak et al., 2014; Omidvar et al., 2018). However, 5.7% of girls had their menarche between 9 and 11 years, which draws concern in view of a reported increased risk of breast cancer (Maria et al., 2014; ACS: Breast Cancer facts and figures, 2019), and needs to be studied with a larger population sample of girls in Meghalaya. 

Several studies in India and other LMICs demonstrated that the knowledge of breast cancer and BSE practice positively relates with woman’s own or family’s socioeconomic status, educational level, employment status, or being married (Okobia et al., 2006; Khokhar, 2010; Shankar et al., 2017; Sharma et al., 2013). It is surmised that some of these categorical variables may have a cause and effect relationship or maybe interdependent in the surveyed populations. For example, educational level and employment status are synonymous with each other in most situations. Similarly, a comparison between married and unmarried may have the age and maturity factor influencing the result. We included questions on father and mother’s educational status in the present survey (see Table 1). However, the analysis of the effect of parents’ education on girls’ knowledge of breast cancer proved to be merely a surrogate reflection of family income. Hence the data is not presented here and instead, a statistically significant relationship between the parents’ education and family income shown in Table 5. The present study, having the advantage of a cohort of girls belonging to a limited age-group, literate with a broader range of continuing educational status and known demographic profile, therefore, appeared most appropriate for studying the impact of current academic level and family income on the knowledge of breast cancer, BSE and its practice as well as the prevalence of known risk factors in the cohort of girls.

Our results show that breast cancer and BSE awareness and the practice of BSE were significantly associated with girls’ education level and family’s monthly income. Thus, it is evident from the data shown in Table 3 that girls in higher academic classes have a greater degree of breast cancer and BSE knowledge, along with the frequency of performing BSE than their fellow-girls in academic courses before them. The family income also impacts similarly; girls from higher income groups better understand breast cancer and BSE knowledge and practice. As shown in Table 4, girls with a higher education level were more consistent with planned physical activity and were less frequently placed in the “never” category. Similarly, girls from a higher income group were steady in physical activity than those from lower income groups. In contrast, consuming high fat/oily food is inversely related to girls’ educational level indicating that girls with higher educational attainments were less inclined to take oily food regularly. This tendency had no impact of the family’s economic status. Results also show a positive relationship between alcohol drinking and girls’ educational level and the family’s economic level, suggesting that girls with higher education and financial background were more disposed towards alcohol drinking. Our results did not find any significant relationship between the girls’ BMI and their educational level and a weaker relationship with family’s financial status (Table 4).

In conclusion, it evident that there exists a prevalent lack of knowledge on breast cancer and the practice of breast self-examination among senior school and college-going girls in Shillong. Therefore, it is suggested that Meghalaya’s government initiates a public health educational intervention to educate young women through culturally relevant mass and social media, lectures, and demonstrations in educational institutions on the risk factors of female breast cancer and the importance of BSE. Further, dedicated facilities may be created for breast cancer early diagnosis in the state’s public health system.
